# Impact of P16 Positivity on Clinical Outcomes in Nasopharyngeal Carcinoma: A Single Institution Study

**DOI:** 10.7759/cureus.35308

**Published:** 2023-02-22

**Authors:** Bipin Ghimire, Emma Trentacosta, Shrinjaya Thapa, Ujjwal Karki, Anusim Nwabundo, Can Wang, Shyam K Poudel, Julie George, Ishmael Jaiyesimi

**Affiliations:** 1 Internal Medicine, Beaumont Health, Royal Oak, USA; 2 Hematology and Oncology, Beaumont Health, Royal Oak, USA; 3 Biostatistics, Beaumont Health, Royal Oak, USA

**Keywords:** overall survival, progression-free survival, immunohistochemistry, p16, nasopharyngeal cancer

## Abstract

Introduction

Nasopharyngeal carcinoma (NPC) is a rare malignancy with unique geographical distribution. It is prevalent in East and Southeast Asia and rare in non-endemic countries like the USA. P16 is a tumor suppressor gene and there are limited studies with inconsistent results describing the association of its positivity in immunohistochemistry and clinical outcomes. In this retrospective study, we compared progression-free survival (PFS) and overall survival (OS) based on p16 positivity in 60 patients with NPC.

Materials and methods

Patients aged above 18 years and followed between July 2015 and December 2020 were included in the study. P16 positivity was based on the immunohistochemistry of the biopsy sample. We compared PFS and OS among all p16-positive and negative patients, and then among patients with advanced disease (stage III or IV), and between p16-positive, negative, and unknown status patients.

Results

There were 15 p16-positive, and 28 p16-negative, with a median age of 54.3 years and 55.7 years respectively. Most patients in both groups were male, Caucasian, and had advanced disease (stage III or stage IV). Both median PFS (p=0.838) and OS (p=0.776) were 84 months in the p16-negative group but were not reached during the study period in the p16-positive group. Among advanced-stage patients, the PFS (p=0.873), and OS (p=0.773) of both groups were not statistically significant. P16 status was unknown for 17 patients, and PFS (p=0.785) and OS (p=0.901), when compared among patients with p16-positive, negative, and unknown status, were also statistically non-significant.

Discussion and conclusion

Our analysis suggests that p16 status does not predict clinical outcomes in NPC patients. Our sample size was limited but is larger than most studies describing this association. With different studies in the literature reporting disparate findings, we recommend larger prospective studies to better illustrate the impact of p16 positivity on clinical outcomes in NPC.

## Introduction

Nasopharyngeal carcinoma (NPC) is a rare malignancy with unique geographical distribution. It is prevalent in East and Southeast Asia and is rare in non-endemic countries, with a reported incidence in the USA being <1 in 100,000 population [1]. Studies in the past have highlighted a few prognostic factors for NPC, of which, advanced age and stage are the most common poor prognostic indicators [2]. Pre- and post-treatment Epstein Barr Virus (EBV) DNA levels are also presented as a potential marker for prognosis and disease recurrence [2,3].

P16 is a tumor suppressor gene, and its immunohistochemistry has a well-defined role in many clinical scenarios [4]. It is also used as an indicator of human papillomavirus (HPV) infection [4]. Its expression is considered an independent prognostic factor regardless of HPV positivity in oropharyngeal carcinoma, with better outcomes in patients with p16 overexpression [5,6]. Literature for its use in NPC, independent of HPV status, is rare. Limited studies have described this association, and there have been inconsistent results among them, with some reporting favorable outcomes in p16-positive patients [7-10], and some without any significant difference [5,11,12].

Jiang et al. reported improved progression-free survival (PFS) with p16 overexpression in EBV-positive patients [7]. Matikite et al. reported an inferior survival rate in patients with a complete absence of p16 expression [8]. Rosalez-Perez et al. described significant development of distant disease recurrence with p16 under-expression [9]. Wilson et al. concluded that p16 was a strong predictor of outcomes in all pharyngeal cancers but was not significant when stratified for NPCs [5]. Even though Sun et al. described a higher risk of lymph node metastasis, and distant metastasis with p16 under-expression but no changes in PFS or overall survival (OS) was noted [11].

In this study, we describe the association between p16 and disease outcomes by comparing PFS and OS in NPC patients based on p16 positivity.

## Materials and methods

Study design and patients

This is a retrospective observational study approved by the Beaumont Health System’s Institutional Review Board (IRB #2019-007). We reviewed charts of patients aged 18 years or above, diagnosed with NPC, and who were followed in the hospital system between July 1, 2015, and December 31, 2020. Sixty patients with NPC were reviewed, and included in the analysis.

Variables and data collection

All data regarding patient characteristics and outcomes were collected using the hospital’s electronic record system. The cancer registry was also utilized to determine the date of death for some patients. Patient characteristics including demographics (age, gender, race), smoking, alcohol intake, family history of NPC, and stage of disease at diagnosis were collected. Data regarding p16 status was then determined based on the pathology report. Outcomes of interest were PFS and OS. Death rate and rate of disease progression were also calculated. Disease progression was defined as the development of new cancerous lesions or an increase in the size of current lesions, diagnosed by either imaging or biopsy. For patients with only a hospice enrollment date available and no date of death on file, the date of death was assumed six months after the hospice enrollment date.

P16 Status

P16 expression in a biopsy sample was detected via immunohistochemistry and was reported as positive when the sample was stained with fluorescence.

Statistical analysis

Outcomes of interest were compared between two groups: p16-positive and p16-negative patients. Categorical variables are reported as either frequencies or percentages. They were examined with chi-square tests when appropriate (expected frequency >5 in 80% of cells), otherwise Fisher’s Exact tests were used. Continuous variables like age and pack years are reported as median (minimum, maximum). We compared PFS and OS using the Kaplan-Meier curves. PFS and OS were calculated from the day of diagnosis. All statistical analyses were performed using SAS 9.4 (SAS Institute, Cary, NC) software, and the comparison of outcomes was considered statistically significant if the p-value was less than 0.05.

Primarily, PFS, OS, and death rates were compared between the p16-positive and p16-negative patient groups. Secondarily, patients with stage III or IV disease were analyzed separately. PFS and OS were also compared between patients of unknown p16 status with p16-positive and negative patients. Finally, a subset analysis comparing p16 positive and p16 negative patients according to EBV status was also performed.

## Results

Patient characteristics

Among the 60 patients with NPC, 43 had results available for the p16 immunohistochemistry test from the tumor sample.

Out of the 43 patients, 15 were p16-positive and 28 were p16-negative. The baseline characteristics of the patients are presented in Table 1. The median age of diagnosis was similar in both groups (54.3 years vs 55.7 years), and most patients were male. A total of 86.7% of p16-positive patients were Caucasian and no patients were of Asian race. Similarly, most patients in the p16-negative group were also Caucasian (46.4%), but also consisted of 17.9% Asian patients. Most patients were former smokers in both groups (46.7% vs 35.7%), whereas 60% of patients had a history of alcohol intake in the p16-positive group compared to 35.7% in the p16-negative. Only a few patients had a history of heavy alcohol intake (13.2% vs 7.1%). Most patients had either stage III or stage IV disease at diagnosis (86.6% in p16-positive vs 82.2% in p16-negative). EBV DNA was positive from the biopsy sample in 33% and 28% of p16-positive and p16-negative patients respectively. Similarly, EBV status was negative in 46.7% and 35.7% of the p16-positive and negative patients respectively. A total of 100% of p16-positive and 94.6% of p16-negative patients received concurrent chemotherapy as per standard protocol.

**Table 1:** Baseline characteristics among p16-positive and negative patients EBV: Epstein-Barr Virus

Patient Characteristics		P16-positive (N=15)	P16-negative (N=28)
Median Age		54.3 years	55.7 years
Gender	Male	66.7% (10/15)	71.4% (20/28)
	Female	33.3% (5/15)	28.6% (8/28)
Race	Caucasian	86.7% (13/15)	46.4% (13/28)
	African American	13.3% (2/15)	25.0% (7/28)
	Asian	0% (0/15)	17.9% (5/28)
	Others	0% (0/15)	10.7% (3/28)
Smoking status	Non-smoker	26.7% (4/15)	35.7% (10/28)
	Current smoker	26.7% (4/15)	28.6% (8/28)
	Former smoker	46.7% (7/15)	35.7% (10/28)
Alcohol intake	Yes	60% (9/15)	35.7% (10/28)
	No	40.0% (6/15)	64.3% (18/28)
Stage at diagnosis	Stage I	0% (0/15)	0% (0/28)
	Stage II	13.3% (2/15)	17.9% (5/28)
	Stage III	53.3% (8/15)	28.6% (8/28)
	Stage IV	33.3% (5/15)	53.6% (15/28)
EBV DNA status (from biopsy)	Positive	33.3% (5/15)	28.6% (8/28)
	Negative	46.7% (7/15)	35.7% (10/28)
	Unknown	20.0% (3/15)	35.7% (10/28)
Treatment received	Induction chemotherapy	20.0% (3/15)	10.7% (3/28)
	Concurrent chemo-radiation	100% (15/15)	96.4% (27/28)
	Adjuvant chemotherapy	20.0% (3/15)	32.1% (9/28)
	Immunotherapy	6.7% (1/15)	10.7% (3/28)
	Surgery	13.3% (2/15)	3.6% (1/28)

Main results

Initially, patients in all stages of the disease were analyzed together. Patients were followed up for a median duration of 37.3 months, and the total number of deaths was 15 including one patient enrolled in hospice.

The death rate of p16-positive patients was similar to that of p16-negative patients (40% vs 35.7%), and the rate of progression was 26.7% vs 42.9% in p16-positive and negative groups respectively.

**Figure 1:**
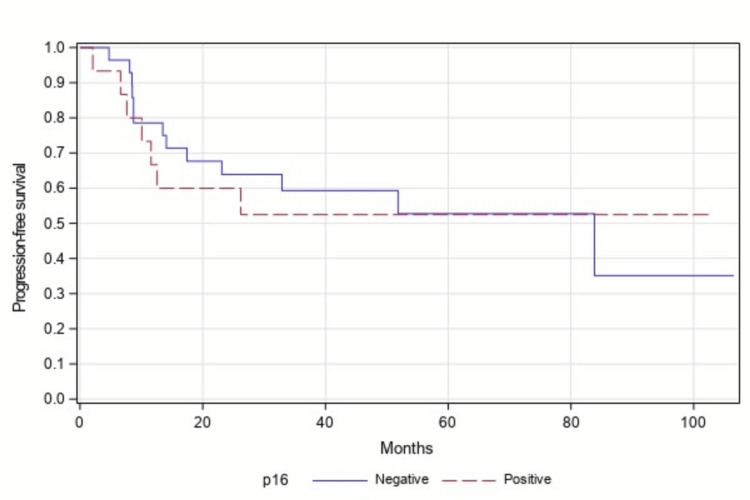
Progression-free survival between p16-positive vs p16-negative patients (n=43), p=0.838

Median PFS was not reached in the p16-positive group and was 84 months in the p16-negative group. PFS was not statistically significant between both groups (p=0.838, Figure 1).

**Figure 2:**
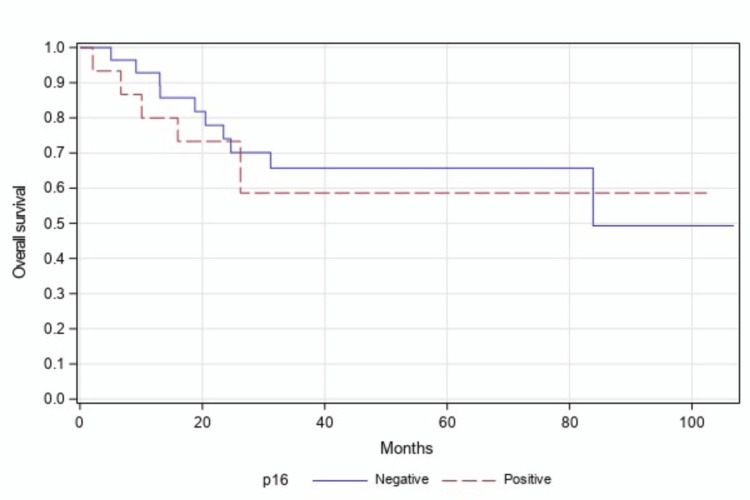
Overall survival in p16-positive vs p16-negative patients (n=43), p=0.776

Similar to PFS, median OS was not reached in the p16-positive group and was 84 months in the p16-negative group. The analysis did not show any statistical significance between the OS of both groups (p=0.776, Figure 2).

**Figure 3:**
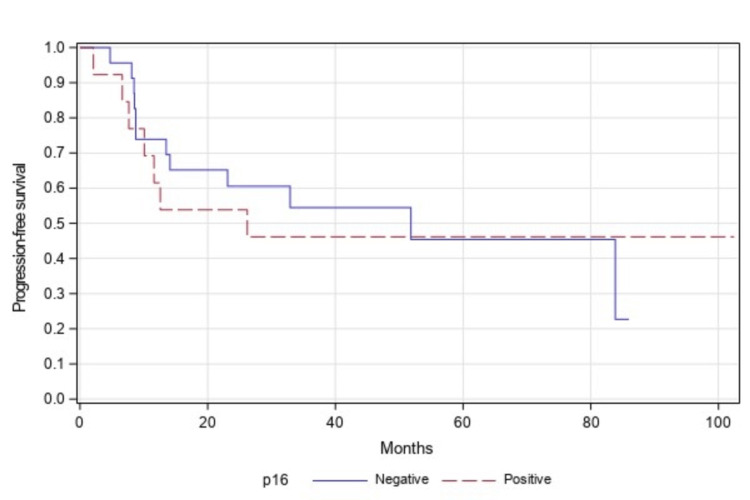
Progression-free survival of p16-positive vs p16-negative (stage III/IV patients) (n=36, p=0.873)

**Figure 4:**
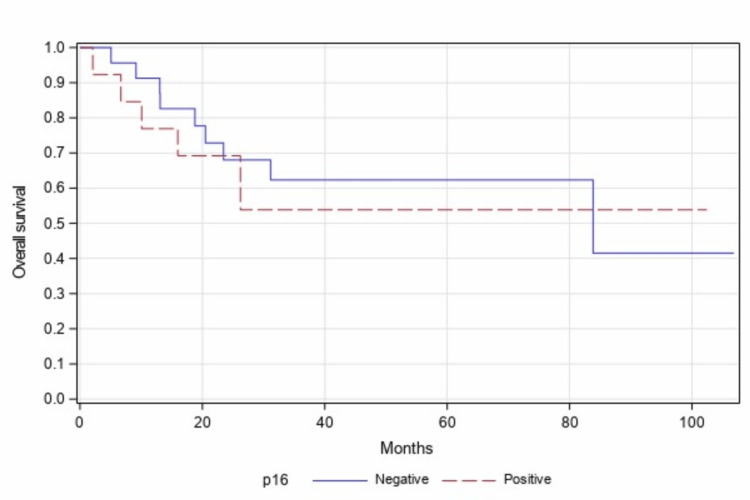
Overall survival of p16-positive vs p16-negative (stage III/IV patients) (n=36, p=0.773)

On further sub-group analysis in patients with only stage III or stage IV disease, similar findings were observed as PFS (p=0.873, Figure 3) and OS (p=0.773, Figure 4) and were not statistically significant in both groups of patients.

Seventeen patients did not have p16 status results available from the tumor biopsy sample. A separate analysis was done including patients with unknown p16 status as a third group and compared with p16-positive and negative patients.

**Figure 5:**
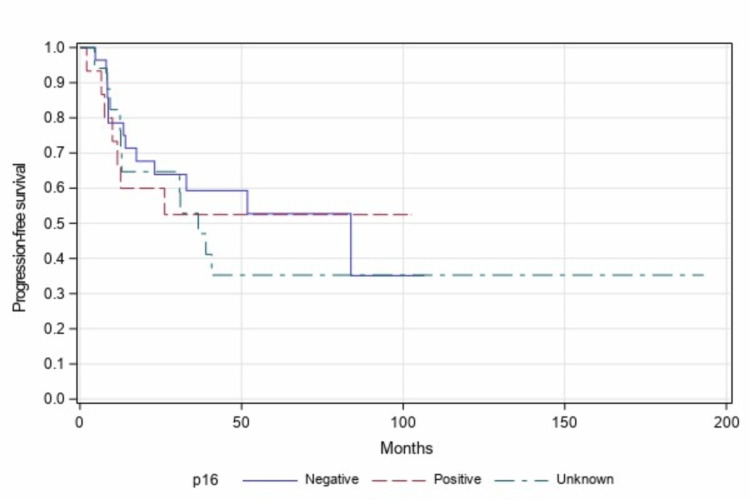
Progression-free survival among p16-positive vs p16-negative vs p16 unknown patients (n=60, p=0.785)

**Figure 6:**
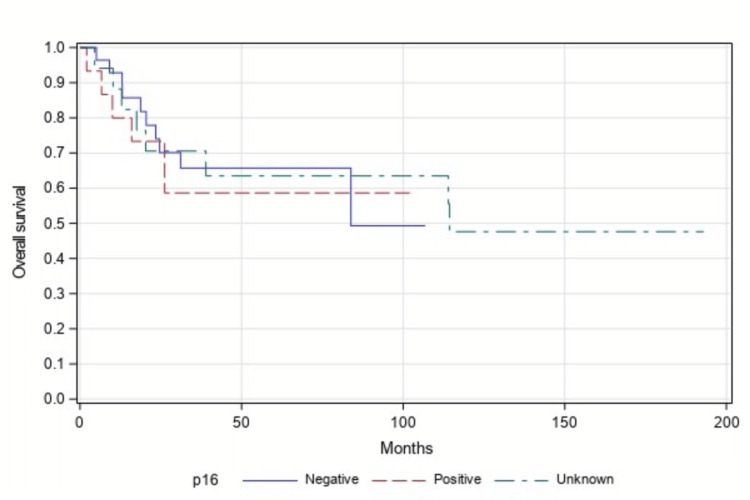
Overall survival among p16-positive vs p16-negative vs p16 unknown patients (n=60, p=0.901)

This analysis was also statistically insignificant when comparing PFS (p=0.785, Figure 5) and OS (p=0.901, Figure 6) in all three groups.

We also compared PFS and OS in p16-positive and negative patients according to EBV status. There were five p16-positive and eight p16-negative patients within the EBV-positive group. Similarly, among EBV-negative patients, seven were p16-positive and 10 were p16-negative.

**Figure 7:**
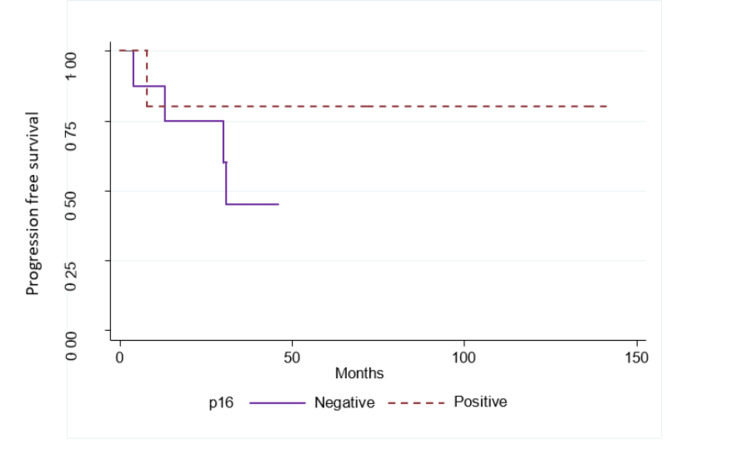
Progression-free survival in p16-positive vs p16-negative (EBV-positive patients) (n=13, p=0.31) EBV: Epstein-Barr Virus

**Figure 8:**
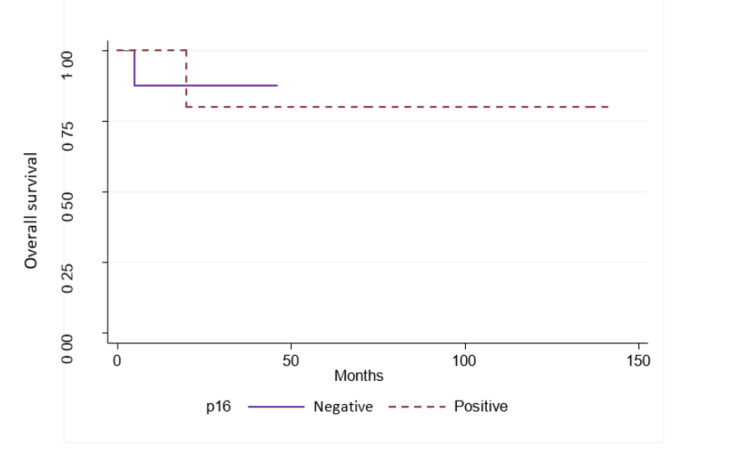
Overall survival in p16-positive vs p16-negative (EBV-positive patients) (n=13, p=0.87) EBV: Epstein-Barr Virus

**Figure 9:**
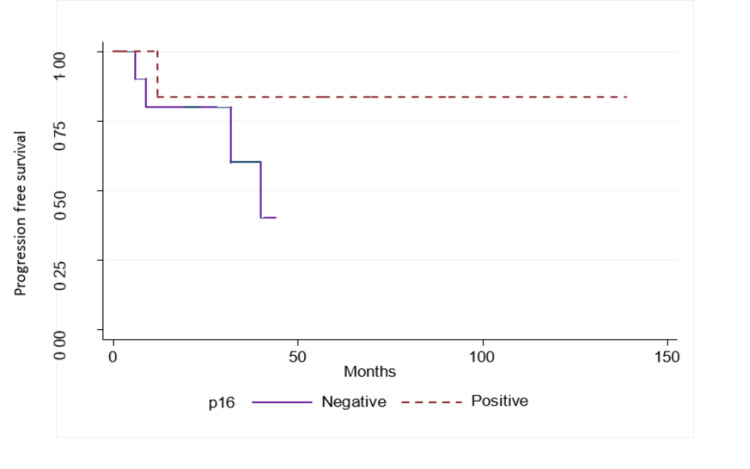
Progression-free survival in p16-positive vs p16-negative (EBV-negative patients) (n=17, p=0.25) EBV: Epstein-Barr Virus

**Figure 10:**
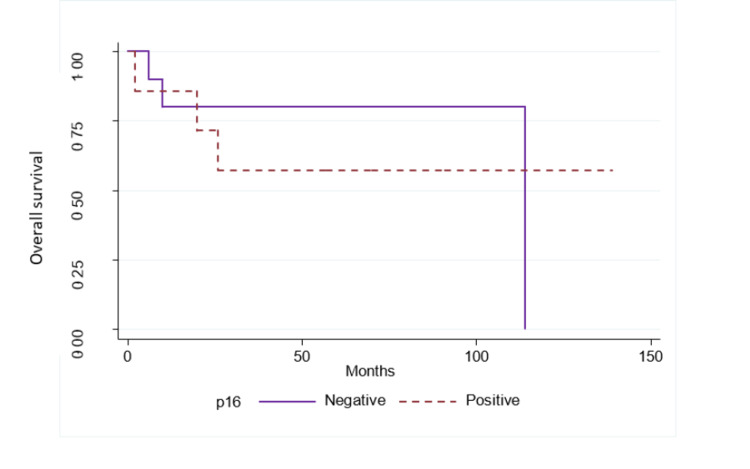
Overall survival in p16-positive vs p16-negative (EBV-negative patients) (n=17, p=0.74) EBV: Epstein-Barr Virus

Among EBV-positive, there was no statistical difference in PFS (p=0.31, Figure 7 in Appendix) and OS (p=0.87, Figure 8 in Appendix) between p16-positive and negative patients. Similarly, statistical difference was not observed among EBV-negative patients in both PFS (p=0.25, Figure 9 in Appendix) and OS (p=0.74, Figure 10 in Appendix).

## Discussion

P16 is a tumor suppressor gene found in many different types of cancers, the second most common after p53, and belongs to the INK4 family [4]. The immunohistochemistry of p16 has a well-defined role in many clinical scenarios. For example, it shows strong nuclear positivity in desmoplastic melanoma and loses expression in malignant mesotheliomas [4]. P16 is also used as an indicator for HPV infection, which can manifest as anogenital lesions, uterine cervical glandular lesions, vulvar lesions, or oropharyngeal squamous cell carcinoma [4].

P16 immunohistochemistry has shown a strong correlation with HPV positivity in oropharyngeal carcinoma [5,13]. However, the p16 correlation with HPV positivity in other pharyngeal cancers, such as NPC, is not well studied. Limited studies have shown p16 to have good sensitivity but low specificity to detect HPV status [5,14]. In our study, we investigated the impact of p16 positivity on PFS and OS in NPC patients. P16 was not used as a surrogate marker for HPV infection, and its impact on disease outcomes was analyzed independent of HPV status.

From our analysis, we suggest that p16 status does not predict disease outcomes with PFS and OS; although the rate of progression was lower in the p16-positive group of patients. Stage of disease is an independent prognostic factor for NPC [2], and there was no difference in survival when advanced-staged patients (III and IV) were analyzed separately. Age is also an established prognostic factor [2]. Even though a separate stratified analysis based on age was not completed, patients in both groups had a comparable median age at diagnosis. EBV is widely accepted as a key factor in the development and progression of NPC [3]. When stratified for EBV status, there was no statistical difference in survival of p16-positive and negative patients; however, the patient population was relatively smaller in each group.

Findings similar to our analysis have been reported in other studies. Wilson et al. studied 13 patients with nasopharyngeal cancer and concluded that p16 positivity was not a significant predictor of outcomes [5]. Fakhry et al. analyzed 15 NPC patients with reported p16 status and did not find any difference in survival due to p16 positivity [12].

Contrary to our findings, few studies have described a positive association of p16 expression with disease outcomes. Jiang et al. reported that p16 overexpression among EBV-positive patients correlated with improved PFS and loco-regional control [7]. Makitie et al. studied 59 patients with nasopharyngeal cancer and concluded that the complete absence of p16 expression was associated with an inferior OS rate [8]. Wang et al. concluded that p16-positive patients had superior survival rates compared to p16-negative patients [15]. A meta-analysis including these three studies, however, failed to show any significant difference in clinical outcomes (OS, PFS) based on p16 positivity [11]. However, it did demonstrate higher distant metastasis, lymph node metastasis, and TNM staging in patients with down-regulated p16 expression [11]. Rosalez-Perez et al. also described an aggressive clinical course in p16-negative patients who had metastases present at diagnosis [9]. Although they did not report disease outcomes, Zhang et al. [16] and Fan et al. [17] found loss of p16 expression was significantly increased in NPC tissues compared to normal nasopharyngeal epithelial cells. Lin et al. hypothesized that loss of p16 expression might confer resistance to NPC treatment as they found 96% of patients requiring salvage therapy had a loss of p16 expression [10].

There are some limitations to our study. First, this was a single-institution study with a relatively small sample size. Some studies have also described race as a potential factor that can impact survival, but this is not well established [2,18]. Among all patients in our study, only 11.6% were Asian, all of whom were p16-negative. With our sample size, we were not able to conduct a separate analysis based on race, but we believe this difference possibly is not large enough to skew the overall outcomes of the study. Secondly, our study sample included patients with unknown EBV DNA status from the tissue biopsy as depicted in Table 1. We did not find a statistical difference in the survival of p16-positive and negative patients among those with known EBV status, however, the smaller subset of these patients might limit the analysis.

Lastly, our study population also included 17 patients whose p16 status was unknown. A separate analysis comparing all three groups (p16-positive, negative, and unknown status) did not show a significant difference in both OS or PFS. It would be helpful if we had known the p16 status of the 17 patients. If a majority of those patients belonged to either the p16-positive or negative group, this could possibly change the outcome of our study. Although this is a possibility, we cannot deny the fact that our sample size of patients with known p16 status was larger than most studies that have investigated this association.

Considering multiple studies have shown contradicting results, larger prospective studies are required to better describe the association between p16 positivity in NPC patients and their clinical outcomes.

## Conclusions

In summary, this is a retrospective study of a single institution evaluating outcomes of NPC patients based on p16 positivity. Forty-three patients had known p16 status with a median follow-up of 37.5 months. There was no difference in progression-free and overall survivals between p16-positive and negative patients. Similarly, there was no difference in the progression-free and overall survivals for patients with advanced stage III and stage IV disease, and when stratified for EBV status. Taken together, this study suggests that p16 status in isolation does not predict outcomes in NPC patients. With disparate findings on the association of p16 and NPC in published literature, our results are hypothesis-generating and need larger prospective studies to better illustrate the impact of p16 status on clinical outcomes in patients with NPC.

## References

[1] Argirion I, Zarins KR, Ruterbusch JJ, Vatanasapt P, Sriplung H, Seymour EK, Rozek LS (2020). Increasing incidence of Epstein-Barr Virus-related nasopharyngeal carcinoma in the United States. Cancer.

[2] Bossi P, Chan AT, Licitra L (2021). Nasopharyngeal carcinoma: ESMO-EURACAN Clinical Practice Guidelines for diagnosis, treatment and follow-up(†). Ann Oncol.

[3] Alami IE, Gihbid A, Charoute H (2022). Prognostic value of Epstein-Barr virus DNA load in nasopharyngeal carcinoma: a meta-analysis. Pan Afr Med J.

[4] Serra S, Chetty R (2018). p16. J Clin Pathol.

[5] Wilson DD, Crandley EF, Sim A (2014). Prognostic significance of p16 and its relationship with human papillomavirus in pharyngeal squamous cell carcinomas. JAMA Otolaryngol Head Neck Surg.

[6] Rosenthal DI, Harari PM, Giralt J (2016). Association of human papillomavirus and p16 status with outcomes in the IMCL-9815 Phase III registration trial for patients with locoregionally advanced oropharyngeal squamous cell carcinoma of the head and neck treated with radiotherapy with or without cetuximab. J Clin Oncol.

[7] Jiang W, Chamberlain PD, Garden AS (2016). Prognostic value of p16 expression in Epstein-Barr Virus-positive nasopharyngeal carcinomas. Head Neck.

[8] Mäkitie AA, MacMillan C, Ho J (2003). Loss of p16 expression has prognostic significance in human nasopharyngeal carcinoma. Clin Cancer Res.

[9] Rosales-Pérez S, Cano-Valdez AM, Flores-Balcázar CH (2014). Expression of Epstein-Barr Virus-encoded latent membrane protein (LMP-1), p16 and p53 proteins in nonendemic nasopharyngeal carcinoma (NPC): a clinicopathological study. Arch Med Res.

[10] Lin HS, Berry GJ, Sun Z, Fee WE Jr (2006). Cyclin D1 and p16 expression in recurrent nasopharyngeal carcinoma. World J Surg Oncol.

[11] Sun L, Song J, Huang Q (2019). Clinicopathological and prognostic significance of p16 protein in nasopharynx cancer patients: a PRISMA-compliant meta-analysis. Medicine (Baltimore).

[12] Fakhry C, Westra WH, Wang SJ (2017). The prognostic role of sex, race, and human papillomavirus in oropharyngeal and nonoropharyngeal head and neck squamous cell cancer. Cancer.

[13] Simon J, Schroeder L, Ingarfield K (2020). Epstein-Barr virus and human papillomavirus serum antibodies define the viral status of nasopharyngeal carcinoma in a low endemic country. Int J Cancer.

[14] Stenmark MH, McHugh JB, Schipper M (2014). Nonendemic HPV-positive nasopharyngeal carcinoma: association with poor prognosis. Int J Radiat Oncol Biol Phys.

[15] Wang L, Yao L, Zhang S, Liang C, Cheng N (1999). Relationship between expression of P16 protein and prognosis in carcinoma of nasopharynx [Article in Chinese]. Hua Xi Yi Ke Da Xue Xue Bao.

[16] Zhang L, Fang Y, Huang B, Hou J, Zhao M, Li H, Zeng Y (2002). Rapid tissue microarray assay of p16 protein expression for different stage nasopharyngeal carcinoma. Zhonghua Bing Li Xue Za Zhi.

[17] Fan SQ, Ma J, Zhou J (2006). Differential expression of Epstein-Barr virus-encoded RNA and several tumor-related genes in various types of nasopharyngeal epithelial lesions and nasopharyngeal carcinoma using tissue microarray analysis. Hum Pathol.

[18] Stepan KO, Mazul AL, Skillington SA (2021). The prognostic significance of race in nasopharyngeal carcinoma by histological subtype. Head Neck.

